# Computational Psychiatry for Computers

**DOI:** 10.1016/j.isci.2020.101772

**Published:** 2020-11-07

**Authors:** Eric Schulz, Peter Dayan

**Affiliations:** 1 Max Planck Institute for Biological Cybernetics, Tübingen, Baden-Württemberg, Germany; 2 University of Tübingen, Tübingen, Germany

**Keywords:** Computer Science, Human-Computer Interaction, Psychology

## Abstract

Computational psychiatry is a nascent field that attempts to use multi-level analyses of the underlying computational problems that we face in navigating a complex, uncertain and changing world to illuminate mental dysfunction and disease. Two particular foci of the field are the costs and benefits of environmental adaptivity and the danger and necessity of heuristics. Here, we examine the extent to which these foci and others can be used to study the actual and potential flaws of the artificial computational devices that we are increasingly inventing and empowering to navigate this very same environment on our behalf.

## Graphical Abstract

**Figure:**
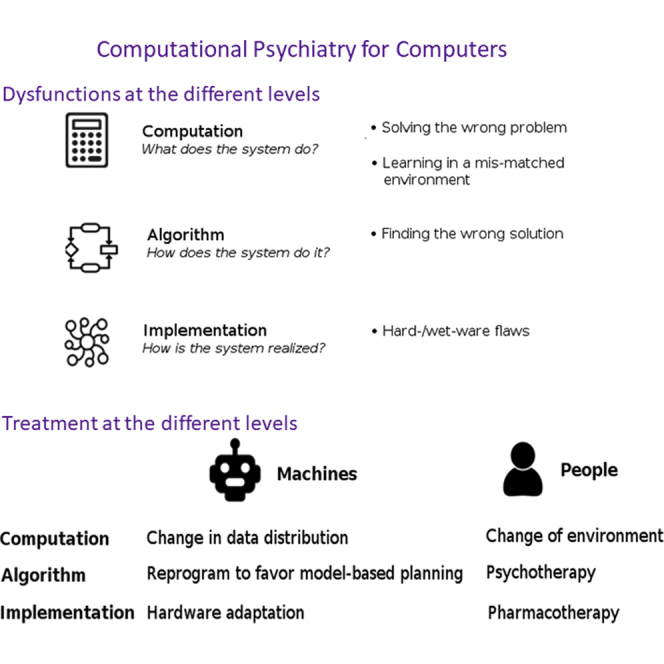

To err is human, but really to foul up takes a computer.

—Paul R. Ehrlich

## Introduction

To err is human. We can be irrational, illogical, ignorant and irresponsible, and our actions and decisions can lead to irredeemable harm to ourselves and others. When such behaviors are extreme relative to societal norms, and persistent even in the light of evidence of the attendant damage, they are often considered dysfunctions. We then enter the medical realms of neurology and psychiatry, which, put very crudely, consider breakdowns respectively in the neural and psychological architectures of thought, feeling and action and the way these are underpinned by learning and adaptation.

However, there is an increasing realization that the manifold flaws that afflict even the healthy have their roots in what is a fundamental and foundational problem of existence. We have to make choices in an environment replete with threats as well as opportunities, but of which, because of both initial uncertainty and change, we are only rather dimly aware. Performing perfectly, or in many cases even well, over the long run in such circumstances is radically computationally intractable. Thus, approximations and heuristics, which by their very nature can never lead to perfect performance in all circumstances, are inevitable. How then should we think of the dysfunctional escalation of these problems in psychiatric and neurological disorders?

The field of *computational psychiatry* (CP) adopts this perspective as its leitmotif (Montague et al., 2012; Huys et al. 2016). CP considers the interactions between individuals, populations of individuals and evolutionary, developmental and current environments that collectively define good- and bad-quality choice. It then attempts to use these to provide insights into the nosology, prognoses and even possible cures for some of the aforementioned flaws.

Here, along with, for instance, Mainen (2018), we argue that there is a further unavoidable consequence of this perspective that applies to sufficiently complex systems of any sort making decisions in similarly such complex environments. These systems exactly include the newly powerful agents developed in modern artificial intelligence and machine learning, which are being applied to domains spanning object recognition, speech recognition, and control, and to which we are increasingly delegating authority and autonomy. The unavoidable fact is that such systems will also be faced with trade-offs which, while they might differ in detail from ours because of the different capacities of their idiosyncratic computational implementations, will nevertheless run into the same theoretical buffers, and so the same opportunities for bad-quality behavior. Understanding this is essential for us to control their actions, reap their benefits, and minimize their harm (Rahwan et al., 2019).

We therefore propose to turn the lens of CP onto computers themselves to help us to illuminate their systematic failures. We also hope that a CP for computers will enrich our understanding of human psychiatry, for instance by telling us which symptoms are universally associated with computational and statistical complexity, and which are idiosyncratically wet and dry.

We first define what CP attempts to understand and show how its scope can be expanded to analyze machine behavior. We then consider several criteria for psychiatric symptoms in computers and some key differences with human psychiatry. Finally, we make some remarks on the perspective that this analysis affords over treatment.

One important caveat is that CP is itself only in its earliest days, and its foundations, formulation, and future utility are all incompletely certain. A second caveat is that we follow CP's broad program in focusing first on maladaptive decisions, rather than the accompanying and separate emotions and feelings which are critical in many aspects of human psychiatry. Emotions are certainly of great interest in CP (e.g., Bach and Dayan 2017; Sen et al., 2019). However, given the active debate about the status of emotions even in non-human animals (LeDoux 2014; Paul et al., 2020), we pragmatically, if pusillanimously, punt.

## Computational Psychiatry

CP locates symptoms and causes for dysfunctional decision-making in diverse possible breakdowns in the architecture of adaptive choice. We therefore start from a brief description of this architecture and its potential vulnerabilities. This sets the stage for understanding potential flaws in natural and artificial agents.

**Figure 1:**
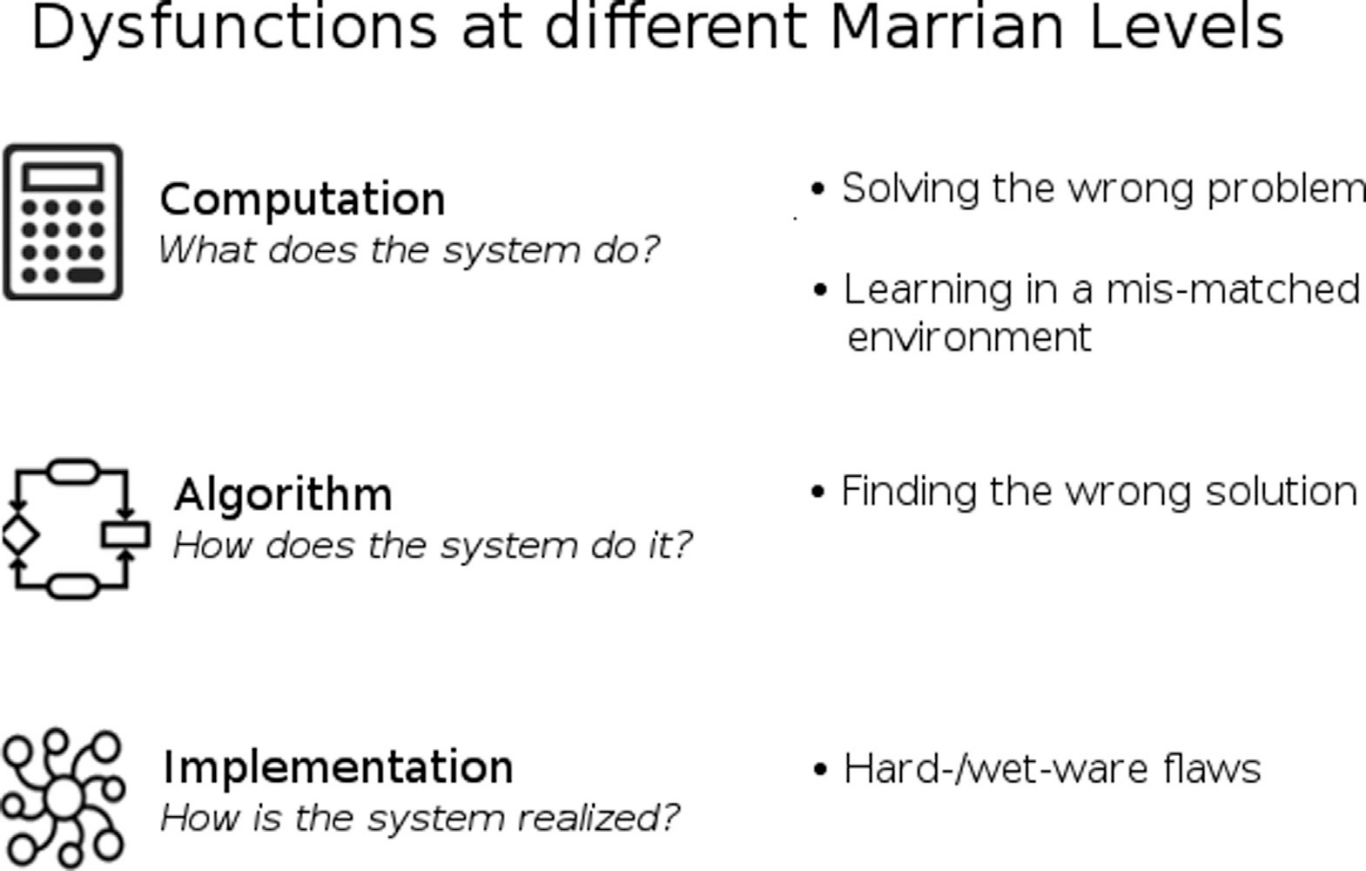
Marr's Levels of Analysis and the Nature of Possible Flaws that Can Occur at Each Level

The architecture can be usefully described at different levels of computational and biological analysis. We adopt the famous division of Marr (1982) (see also Peebles and Cooper 2015; Hamrick and Mohamed 2020; Hauser et al., 2016) into computational, algorithmic and implementational levels (Figure 1). The computational level concerns the tasks the system is trying to solve –here, making choices that maximize survival over the long run in an only partially known and changing environment replete with threats. At this level, we also treat ethological considerations of how systems are fit for the niches they occupy. The algorithmic level concerns the nature of the solutions – e.g., the manifold methods of representing observed and latent aspects of the environment, evaluating options, and thereby making choices. Here, psychological processes characterize the effective procedures that are executed. Finally, the implementational level concerns the physical realization of the solutions – in whatever neural, semiconductor or other computational substrate is relevant. Challenges for natural or artificial decision-making systems can often be most parsimoniously described at specific of these levels, so keeping them at least conceptually distinct is important. We shall see, nevertheless, that they become intertwined in rather particular ways – for instance – inevitable algorithmic incompetence can force us to consider merely satisfactory or boundedly optimal computations (Gershman et al. 2015).

### Computation

Perhaps the most general computational level formalization is *Bayesian Decision Theory* (Berger 2013). This is a comprehensive account of evaluative choice, according to which an agent must consider states, utilities, and actions. The state of the environment is a summary of everything about it that the agent knows and is important for predicting what will happen in the future. The agent has to infer the state from its prior beliefs and past and current observations. The agent should then make a prediction of the long-run future utility that is expected to accrue based on their possible actions. Finally, it should choose the action associated with the optimal expected future utility.

This decision-theoretic characterization of the task faced by the agent already points to failure modes – essentially – the agent could be engaged in solving what amounts to the *wrong problem* (Huys et al., 2015). For instance, it could attempt to maximize an objective function that leads to behavior that could possibly hurt itself or others. This can happen even if all complexities of the decision-making problem have been taken into account. Examples of this type of error are plentiful in science fiction. Isaac Asimov's robotic stories contain various versions of artificial agents who come to behave in unintended ways whilst still notionally obeying the “three laws of robotics”. In Stanley Kubrick's movie “2001: A Space Odyssey”, the artificial intelligence HAL is worried about the completion of its mission to go to Jupiter in case it gets shut down and therefore attempts to kill the mission's crew members.

A more contemporary example of solving the wrong problem comes from Bostrom (2003), who proposed a thought experiment about a “paperclip maximizer”. Here, an artificial general intelligence is supposed to maximize the number of paperclips that are produced. This maximization problem is chosen deliberately, because it is unlikely to ever be implemented and – at first sight – appears to be relatively harmless. However, Bostrom argues that following such a reward function to its logical extreme will lead the AI to transform “first all of earth and then increasing portions of space into paper clip manufacturing facilities”.

What these examples show is that the utility function of artificial agents has to be carefully constructed if one wants to avoid unintended consequences. Arguably, similar mechanisms can also be found in aberrant human behavior. For instance, one potential source of anxiety is concern about either the chance or consequence of catastrophic negative events. Solving the problem of avoiding these can lead to caution that other people might consider to be unreasonable (Bishop and Gagne, 2018).

A rather separate collection of problems – the *wrong environment* (Huys et al., 2015) arises when agents are either programmed (by evolution or design), or learn, to accomplish a task in one environment, but are thereby poorly adapted to solve new tasks when that environment changes. In machine learning terms, their choices and learning embody very strong actual, or inductive, biases. This has the huge advantage of allowing good behavior to arise immediately or after small amounts of learning – something at which natural systems excel. However, problems arise when those biases are inaccurate.

One example of this is Pavlovian misbehavior (Breland and Breland 1961; Dayan et al., 2006). Here, agents have built-in or default propensities (barely mutable inductive biases, to use the terminology in machine learning) that can work against their best interests in particular tasks. For instance, (Hershberger, 1986) built an apparatus in which if chicks approached a cup of food, it would move away at twice the speed, whereas if they moved away from the food, it would move toward them. The chicks have an automatic, default policy to approach and engage with food and its predictors. Although one can imagine good reasons why this might have evolved, in this particular task, it is inappropriate. Human subjects exhibit similar maladaptive behaviors (Guitart-Masip et al., 2014).

A second example of this has become a classic, though potentially apocryphal cautionary tale in machine learning (Dreyfus and Dreyfus 1992). Apparently, researchers in the 1960s trained neural networks on photos of camouflaged tanks and forests. However, because the photos of the tanks had been taken on cloudy days, while photos of plain forest had been taken on sunny days, the neural network learned to distinguish cloudy days from sunny days, instead of – as intended – distinguishing camouflaged tanks from empty forest. Similar problems arise in modern machine learning – as so-called covariate or data set shifts (Quionero-Candela et al., 2009). Equally, when Dasgupta et al. (2019) analyzed the behavior of machine learned representations of sentence embeddings methods, they found that these models frequently thought that one sentence was a negation of another sentence simply because it contained an additional word: although wrong in general, this was true in the training data set because negations of sentences mostly contained an additional “not”.

Particularly pernicious cases of the wrong environment involve path-dependencies in the course of adaptation to environments (Dayan et al., 2018), whereby initial differences can lead to large and persistent divergence in outcomes later on. For instance, it has been observed that children tested in a delayed gratification task with reliable later rewards waited considerably longer for the eventual rewards than children tested in unreliable conditions, and it has been suggested that this could explain why the ability to delay rewards depends on early life events (Kidd et al. 2013). However, imagine a child that goes from an unstable to a stable environment. If the child remains impulsive in the way we have described – indeed appropriately for the original environment – the opportunity to discover that the environment has changed so that it has become more reliable might never be discovered. Such path-dependencies, also prominently suggested for learned helplessness (Maier and Seligman 2016) can bake maladaptive behavior into choice in a rather rigid and fixed manner. Being adaptive to the original environment is essential to be adequately flexible; but it comes at a large potential cost of getting stuck. Modern machine learning to learn algorithms that implicitly learn about priors by repeatedly encountering tasks drawn from a distribution (Thrun and Pratt 1998; Clune 2019) face similar concerns. For example, there is clear evidence that machine learning algorithms can acquire biases that result in detrimental predictions (Gianfrancesco et al., 2018) and unfair decisions (Dwork et al., 2012) with severe real-life consequences.

### Algorithm

Unfortunately, although Bayesian decision theory is descriptively simple, it is computationally penal. This is the source of inevitable algorithmic incompetence – agents are finding the *wrong solution* even if they are trying to solve the correct problem (Huys et al., 2015). This can afflict all the components described. First, take states – these have two aspects: the objective state of the environment and the agent's subjective representation thereof. Although the first is the full description of the decision-making problem at a given time point, the latter can be expressed as a probabilistic Bayesian summary of the agent's knowledge of this description. The agent constantly has to perform inferences about the current state of the environment, thereby integrating its own beliefs about the environment, i.e. its evolving priors, with the incoming data, (known as the likelihood). Since an agent rarely knows its state precisely, it has to maintain a distribution over its uncertainty – this is often wildly intractable.

How does the brain cope with these inferential complexities? One general idea is that it embodies various shortcuts or heuristics (Tversky and Kahneman 1974; Gigerenzer and Selten 2002) that perform approximate computations. For instance, it might perform a very limited form of inference (Lieder and Griffiths 2019; Vul et al., 2014) and thereby trade-off computational sloth for inferential imprecision. This notion can explain several cognitive shortcuts in human decision-making: because people halt their mental computations earlier to safe time and energy, the resulting inferences can then be systematically biased (Dasgupta et al. 2017). Even with their vastly more powerful calculational tools, artificial agents suffer from the same formal problems and so can be biased in the same way.

The second algorithmically problematic component of Bayesian decision theory is the prediction of long-run utility associated with a choice. Optimal behavior needs to weigh the future against the present. For example, as in the example of delayed gratification mentioned above, a brief moment of positive outcomes can lead to dire consequences and, although tempting, might better be avoided; on the other hand, experiencing a small loss now can lead to greater gains later on. Thus, even in the rare circumstances where inference about the present is perfectly possible, the agent faces an additional set of issues in making estimations and predictions that are correct over the long run.

The field of reinforcement learning (Sutton and Barto 2018) includes a number of algorithms that learn how to calculate these estimates, and which can operate singly or in combination. One prominent dichotomy is between so-called model-based and model-free reinforcement learning methods (Daw et al. 2005). Model-free methods learn directly, from experienced rewards and punishments, and without building an explicit model of the environment, to estimate the future utility consequences of different actions. These direct estimates are immediately available; however, learning values that are correct over the long run is statistically challenging, and so model-free predictions are rather inflexible to changes. This means, for instance, that model-free agents might perseverate with actions even when they have experienced that they are no longer appropriate – an evident dysfunction. By contrast, model-based methods construct and use models of the environment. They estimate expected long-run utilities by previsioning, i.e., predicting forward using the models. This property makes model-based choice very flexible – as soon as the agent knows that some characteristic of the environment has changed, this can influence its behavior, even many steps before the characteristic will arise. Unfortunately, calculating long-run estimates using the model is time-consuming, and can place a large burden on working memory, a resource in very short supply in humans at least. Research on the trade-off between model-based and model-free learning has revealed that several psychiatric diseases can be linked to an overexertion of either of the two systems (Gillan and Daw 2016; Voon et al., 2017); and there is speculation that interactions between the systems, with samples drawn from the model being used to train model-free mechanisms (Sutton 1991; Mattar and Daw 2018) could themselves be associated with psychiatric conditions involving issues such as rumination (Gagne et al. 2018). The differing computational and statistical characteristics of model-based and model-free methods imply that artificial systems, just like natural ones, should optimally include both. Thus, artificial systems can suffer the same problems when these systems are misapplied.

### Implementation

Implementational issues, e.g., frank hard- or wet-ware flaws, although critical for the nature and some classes of treatment of dysfunction, are obviously more divergent between humans and machines. We should note, however, that some implementational details span the levels of analysis in a deleterious manner. For instance, one common initial mode of action of very many drugs of addiction appears to be their ability to hijack the normal mechanisms by which the brain reports computationally specific aspects of reward or utility (Redish et al. 2008) to influence current and future behavior. We have already discussed how utility functions can lead to maladaptive choice – effects of drugs on these mechanisms can lead to some similar problems which, in humans and other animals, are then unfortunately exacerbated by other effects of the drugs.

## A Diagnostic Manual of Disorders for Computers?

Our essential argument and examples so far have suggested that many of the failure modes of humans that are characterized in computational and algorithmic terms extend to artificial complex systems operating in the same environments. Machine evidently escape some of our flaws (notably severe limits to processing speed and memory, fatigue, boredom and aging) but could current computers suffer from problems that we do not? To put this another way, the famous Diagnostic and Statistical Manual of Mental Disorders (DSM5) (American Psychiatric Association, 2013) is the latest incarnation of a series of attempts to codify human mental dysfunction. Thus what might one imagine encountering in a DSM5 for computers, or DSM5c?

Of course, one of the main premises of CP's evolving approach (Stephan et al., 2016) to nosology, or the decomposition of mental dysfunction, is that the underlying, largely statistical, categories in DSM5 need enriching with the sort of computational structure that we provided above. Thus, a DSM5c could start off on firmer foundations. However, we conjecture that the conjoint complexity of the architectures of our computers and the amorphous data used to train them, mean that at least some elements of the statistical construction of DSM5 (i.e., the basis it provides for mutually agreed diagnosis) will remain.

One large source of problems for computers stems from the rather overly *tabula rasa* and structurally impoverished nature of many current methods in machine learning, leading to a set of issues that have been well discussed elsewhere (Lake et al., 2017). In a way, these are the flip side of not suffering from the Pavlovian misbehavior we briefly described above (Dayan et al., 2006).

A second source of issues in which machines are currently rather wanting concerns robustness. Brains are extremely robust to the turnover, damage and even destruction of many of their components. By contrast, machines are typically much more vulnerable; it therefore comes as no surprise that increasing the robustness of machine learning algorithms (Feurer et al., 2015), as well as building robots that can cope with damage (Cully et al., 2015) is an exciting topic of ongoing research.

A potentially different aspect of robustness concerns priors. Consider what happens when very unlikely but impactful events occur. For computational decision-making systems, it can be complicated to adjust their internal models in the face of these so-called “black swan” events (Taleb 2007), which live outside previously plausible ranges. By contrast, people (at least when not suffering from anxiety; Gagne et al. 2018) are often more able to update their internal models. One example of this phenomenon occurs in so-called “cautious control” algorithms, which reduce their actions in cases of high uncertainty. This mitigates a common drawback of traditional models, which tend to produce extremely high learning updates in what actions they think are best to perform but then leads to another problem: since these algorithms decrease control with rising uncertainty, this can entirely prevent learning, causing the whole system to turn off during events of high uncertainty (Klenske and Hennig 2016). This is indeed what can happen to algorithmic trading algorithms, which – instead of providing liquidity – can shut down as they detect sharp rises in buying and selling of stocks, thereby intensifying market swings (Easley et al., 2011).

Of course, computers can execute commands orders of magnitudes faster, and over a much larger canvas than human decision-making. This is one of their major strengths and is a main reason why we outsource decision-making to computers in the first place. However, it also means that decision can go wrong far faster, more comprehensively, and at a much larger scale. An example of this effect are the series of vulnerabilities (collectively called “Ripple20”) in a widely used, low-level software library that were discovered in 2020. These vulnerabilites could potentially affect hundreds of millions of devices and put them at risk of attackers to steal their data or modify their devices' source code. The moniker comes from the potential ripple-effect, where a single vulnerable component, although it may be relatively small in and of itself, can ripple outward to impact a wide range of applications, given its widespread usage.

A further area in which machines are currently vulnerable to deficits concerns social factors. We, and many other species of animals, are highly social, and duly enjoy an elaborate, but incompletely understood, collection of social propensities - including such things as socially directed contributions to our utilities (formalizing such factors as envy and guilt and altruism, Fehr and Schmidt 1999; Crockett et al., 2014), learning from imitation and demonstration, emotion contagion (Hatfield et al. 1993), and theory of mind (Frith and Frith 2005). Coming back to the “paperclip maximizer” example, for instance, it is likely that a human decision maker would eventually realize that transforming the whole universe to paperclips, even at the cost of the lives of others, might be a bad idea, and therefore stop before it is too late. Equally, the catastrophic failure of the chatbot Tay to avoid becoming inflammatory and offensive when learning from human interactors shows something of the difficulty machines have in navigating social environments.

Of course, we readily anthropomorphize our computational artifacts (Reeves and Nass 1996). However, current programs rarely incorporate such social factors (perhaps unless explicitly to exploit us). This means that (albeit with important exceptions; (Breazeal et al., 2016) current machines risk exhibiting in interaction with us what would be considered personality disorders of various sorts if exhibited by other humans. This could have substantial attendant costs. One deep-rooted concern is that source verification, that is telling whether or not another agent shares the same reality, is already hard to accomplish for us humans, and perhaps it could even be much worse for computers with their vastly broader and more diverse input base. It would also be interesting to step back and consider the benefits of the cooperation with which we are endowed, for instance with its close ties to culture (Hinde and Groebel, 1991), but that insufficiently social machines lack.

One avenue that has been well explored in machine learning systems is adversarial examples, i.e., suitably minimally altered inputs that cause a network to fail (Szegedy et al., 2013). These can be revealing about the structure of the computation and modes of failure. Although it is putatively such adversarial cases that keep industries such as gambling and social media in their cups, systematic investigations in the case of decision-making are currently thinner on the ground (Dan and Loewenstein 2019; Dezfouli et al. 2020).

## Treating Computers (and People) at Different Level

**Figure 2:**
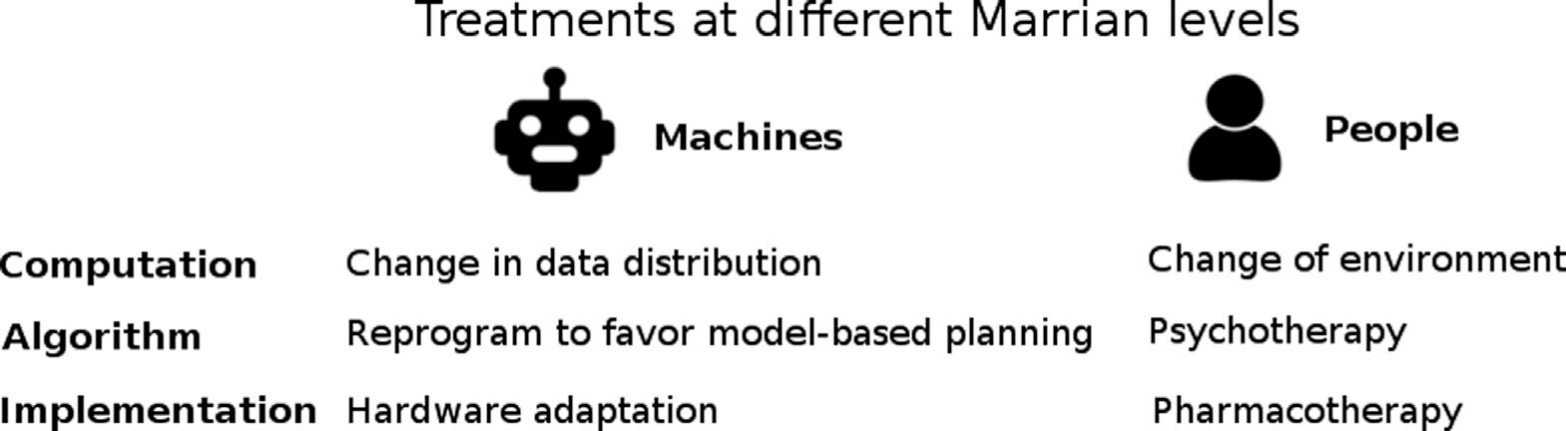
Abstract Examples of Treatment Methodologies at Different Marrian Levels for Machines and People.

Given this CP-based analysis of some of the problems that artificial systems face, what can we say about potential treatments and how we can learn from common practice in human psychiatry? The different levels of computational analysis (see Figure 2) play a more complicated role here – evidenced by the fact that humans might take a systemic drug such as a serotonin reuptake inhibitor, whose direct effects are hard to describe other than implementation, to address a condition that exhibits itself at a psychological/algorithmic level. Indeed, it is perhaps remarkable that the sort of systemic pharmacotherapies that are mainline treatments for humans work at all – given the inevitable severe limits to their specificity of action, and the complex heterogeneity of the brain. The fact that drugs do work invites speculation about modularity and thereby perhaps lead to algorithmic insights (Dayan 2012). Some of these insights might be useful for treating artificial systems too – for instance – if rigidity had set in (perhaps because of an environmental mismatch), then boosting aspects of the way that rewards are processed might provide contrary evidence that resets adaptivity.

In contrast to pharmacotherapy is the machine equivalent of psychotherapy or cognitive behavioral therapy that one might consider directing to the computational and algorithmic levels. At first sight, one might think this to be mildly ridiculous – if we program a device, why are we not in complete, explicit and detailed control of its function. However, if that function is realized by the conjoint values of millions of weights in an artificial neural network, then reprogramming weights directly, though obviously theoretically possible, is a fool's errand – so the tools of a computational psychiatrist are actually surprisingly limited.

Computationally, the question is how to influence utility functions or maladaptive priors about the environment. We have already discussed the difficulty of choosing utility functions that lack unintended consequences – it is very hard to account or legislate for taste. By contrast, empirical priors can often be changed by changing the experience that the agent gets; something that could, for instance, help ameliorate fairness-destroying biases (Corbett-Davies and Goel 2018). This is particularly important for breaking path dependence for reinforcement learning agents that are generally responsible for determining themselves what experience they choose to collect.

The algorithmic level is peopled by heuristics that are typically variable themselves, with parameters determining such things as the relative weighting of model-based and model-free reasoning (which governs flexibility to change), expectations about the potential profitability of the environment (which governs the willingness and structure of exploration), or its volatility (which governs the speed of adaptation and also the speed of forgetting, which can be particularly important for negative events), or its smoothness (which governs generalization from one part to another Schulz et al., 2017). Even when these heuristics are not quite appropriate from a computational level perspective, it is often the case that properties of the environment and/or the agent that are best expressed at the computational level determine good values of the parameters. If these values are incorrect – perhaps because of inaccuracy or over-rigidity, then one can imagine resetting these terms by fiat, thereby generating better behavior and, as with the priors, potentially resetting the computational level to extinguish the maladaptive setting.

## Discussion

CP is a burgeoning field investigating computational differences between healthy subjects and patients who suffer from mental disorders, starting from issues of decision-making. Since problems arise from the radical complexity of the underlying problem, rather than necessarily the fact that biological systems are trying to solve it, we have proposed to use CP to understand artificial systems also (Mainen 2018). The fact that humans and machines are facing similar decision-making problems implies that they can also converge on similar solutions such as developing multiple systems, employing shortcuts, or falling back on hard-wired solutions. Although these solutions can be adaptive, they can also lead to unwanted behavior, where computers can make errors much faster and at greater scale and are less robust to changes in the environment.

Even though these ideas are in their infancy, with much to do even to disentangle the various proposed failure modes in a fully general manner, we suggest that CP for computers offers an additional toolkit of principles and ideas for studying shortcomings and failures in machine behavior. It casts particular light on environmental and developmental issues, as well as fitting and misfitting heuristics. In particular, we intend for this article to motivate researchers to think about and study the similarities and differences between the failure modes of computational and biological systems, eventually leading to a unique and coordinated program of research that will help to to describe, prevent, and treat both sorts. Ultimately, although, we hope that by refining our understanding of failure and success, one of its most important contributions will be to cast new light on ourselves and our unique strengths and shortcomings.
